# Shared Psychotic Disorder with Sexual Delusions

**DOI:** 10.1007/s10508-012-9992-9

**Published:** 2012-07-19

**Authors:** Michal Lew-Starowicz

**Affiliations:** III Department of Psychiatry, Institute of Psychiatry and Neurology, ul. Sobieskiego 9, 02-957 Warsaw, Poland

**Keywords:** Shared psychotic disorder, Folie à deux, Sexual delusions, DSM-IV-TR, ICD-10

## Abstract

Shared psychotic disorder (SPD) is perceived as a relatively rare and poorly understood psychiatric phenomenon. Patients sharing sexual delusions may refer to sex therapists looking for treatment of an alleged sexual pathology. This might cause significant diagnostic and therapeutic challenges. The aim of this article was to discuss diagnostic and management difficulties of SPD with special interest in patients sharing sexual delusions. PubMed selective search was provided for publications with keywords including SPD, induced delusional disorder, folie à deux, and induced psychosis. One case is presented and discussed according to recent diagnostic criteria and the medical and legal issues of the therapy.

## Introduction

Shared psychotic disorder (SPD) or folie à deux, induced psychosis, and induced delusional disorder (IDD) is that which is shared by two or more people with close emotional links. The essence of this phenomenon is a transfer of delusions from one person (inducer) to another (recipient, involved or induced partner). It was first described as folie à deux by Lasegue and Falret in 1877 (Joshi, Frierson, & Gunter, 2006). In the literature, folie à deux is described typically in case reports (Adler & Magruder, 1946; Florez & Gomez-Romero, 2001; Gant & Brown, 2001; Joshi et al., 2006; Oatman, 1947; Petrikis, Andreou, Karavatos, & Garyfallos, 2003; Reif & Pfuhlmann, 2004; Shiwach & Sobin, 1998), so definite prevalence rates are lacking. Generally, it is perceived as a rare condition of unclear etiology, intriguing as a “nature or nurture” phenomenon, and challenging our understanding of the roots of psychopathology. It usually concerns two people (inducer and recipient), rarely three or more (inducer and more recipients) (Dippel, Kemper, & Berger, 1991; Wehmeier, Barth, & Remschmidt, 2003), almost exclusively members of the same family, commonly sisters, husband and wife or parent and child (Gralnick, 1942; Silveira & Seeman, 1995). SPD often causes diagnostic problems and might be hard to distinguish from an independent, endogenous psychosis, especially when found in consanguineous individuals (Dippel et al., 1991; Lazarus, 1985, 1986). Treatment of SPD is complicated due to legal issues and often complimented by a poor response. The presented case of SPD is known to the author who diagnosed and treated the patients at some stage of their illness.

### Diagnostic Considerations

Contemporary diagnostic criteria for SPD according to DSM-IV-TR and induced delusional disorder (IDD) according to ICD-10 are shown in Table 1. Guidelines for diagnosing SPD and IDD do not differ significantly. It is one where only one person suffers from a genuine, established psychotic disorder (inducer), most commonly schizophrenia or delusional disorder. The other person (recipient) is often highly suggestible, younger, less intelligent, more passive, and with lower self-esteem. Both usually live in some kind of isolation from other people combined with a long-standing and very close relationship to each other (Silveira & Seeman, 1995; Wehmeier et al., 2003). Another factor predisposing to receive delusional induction is Dependent Personality Disorder, like in the case of folie à deux and incubus syndrome presented by Petrikis et al. (2003). It is important to distinguish whether the disclosed delusional symptoms are truly psychotic and not representing special cultural beliefs (Gaines, 1995). In affected individuals, psychotic symptoms are clearly believed regardless of evidence to the contrary and usually impair their social, occupational or interpersonal functioning. Distinguishing between the inducer and the recipient can be difficult due to the circular character of IDD where role reversal (when the recipient becomes the inducer) sometimes occurs (Mentjox, van Houten, & Kooiman, 1993). However, in such cases, endogenous etiology in both individuals (more likely when consanguineous) must be ruled out if diagnosing induced psychosis. This was argued by Arnone, Patel, and Tan (2006), who pointed out that the diagnostic criteria of folie à deux are insufficient and do not account for the high rate of psychiatric comorbidity in the recipients. A recent review of court cases involving folie à deux published by Newman and Harbit (2010) showed that the diagnosis of SPD often causes confusion among mental health experts and legal professionals and needs further investigation.

**Table 1:** Diagnostic criteria for Folie à deux-Induced Delusional Disorder in ICD-10 (World Health Organization, 1994) and Shared Psychotic Disorder in DSM-IV-TR (American Psychiatric Association, 2000)

ICD-10	DSM-IV-TR
Induced delusional disorder	Shared psychotic disorder
A. A subject must develop a delusion or delusional system originally held by someone else with a disorder classified in F2–F23	A. Delusion develops in an individual in the context of a close relationship with another person(s), who has an already-established delusion
B. The two people must have an unusually close relationship with one another, and be relatively isolated from other people	B. The delusion is similar in content to that of the person who already has the established delusion
C. The subject must not have held the belief in question prior to contact with the other person, and must not have suffered from any other disorder classified in F20–F23 in the past	C. The disturbance is not better accounted for by another psychotic disorder (e.g., schizophrenia) or a mood disorder with psychotic features and is not due to the direct physiological effects of a substance (e.g., a drug of abuse, a medication) or a general medical condition

### Etiology and Symptoms

The etiology of individual’s delusional symptoms is difficult to evaluate in clinical practice and often remains unknown. Basically, pathogenetic mechanisms involve biological as well as psychological explanations. Main medical etiologies include neurodegenerative and other CNS disorders, vascular, metabolic, hormonal or infectious disease, vitamin deficiencies, intoxications and substance abuse, and medication side effects. Among psychological theories, it is worth to mention psychodynamic theories (delusion as a protective psychological response to different types of stress or conflict), cognitive mechanisms (selective attention to threatening information, jumping to conclusions from insufficient information, and attributing negative events to external personal causes). Delusional symptoms in induced individuals can be based on family and interpersonal dynamics, with tendency of being submissive and other personality traits, or intellectual limitations. Social or geographical isolation, poverty, and language difficulties are additional predisposing contributors (Fennig, Fochtmann, & Bromet, 2005). To sum up, typical patterns of developing induced psychosis include susceptibility (medical and psychological conditions) and being in a very close relationship with a person who suffers from endogenous psychosis and has significant impact on the recipient, connected with relative isolation from environmental influences.

In a study by Appelbaum, Robbins, and Roth (1999), 29 % of acutely hospitalized psychiatric patients were rated as definitely or possibly delusional. The most common types were persecutory (78.4 % of delusional patients), body/mind control (59.5 %), grandiose (43 %), thought broadcasting (35.1 %), religious (28.4 %), guilt (9.8 %), and somatic (9.1 %) delusions. According to the literature, affected individuals most often share delusions of persecution, prejudice, grandiosity, and religious delusions. Sexual content can also be present in delusional thoughts—28 % of psychiatric inpatients with delusions reported some form of sexual delusion (Rudden, Sweeney, Frances, & Gilmore, 1983). However, to my knowledge, no SPD with predominant sexual delusions in non-consanguineous partners has been described until now.

## Case Report

Mr. D, a 64-year old male with higher technological education, a retired academic professor, married for 39 years, was admitted to the Department of Psychiatry with a diagnosis of SPD. He was previously hospitalized for 4 months in another hospital with the same diagnosis, treated with perazine and signed himself out after 12 days. He continued ambulant treatment, changed later to risperidone without any substantial improvement. On his wife’s (Mrs. E, 61 years-old) initiative, before any psychiatric treatment, they had turned for help to a psychologist, an exorcist, and finally to a sexologist (who was also a psychiatrist) who referred them at that time and now once again to another hospital.

Mr. D agreed to hospitalization as he declared he wanted to undergo civil commitment to prevent him from making unfavorable financial decisions connected with being “exploited by a mistress,” The patient and his wife claimed that he would go out at nights to his female lover who would “stimulate him with Viagra and narcotics,” transport him in her car, have sex with him, and finally the patient would wake up at home and not remember anything that had happened. He got all the information of what was going on from his wife that “examined him with drug tests” and offered that he could sleep tied to the bed with handcuffs to prevent going out at night. The woman that was accused to be the mistress was one of the teachers employed with Mr. D in the same university. Mrs. E claimed that she had installed a camera at home and convinced her husband that he was persecuted and tracked by his mistress and he had also had many “contacts” with other women in the meantime.

Mr. D declared that he had “a double life” for 6 years and had been sexually abused and coerced by his mistress “to sign financial documents in her favor” although he could not remember any of these events. He claimed that he was convinced that situation was true because his wife told him so, and he fully trusts her as they have a very strong romantic relationship. Talking to his wife, he came to the conclusion that he was mentally ill and needed professional care and now even a civil commitment. Mrs. E claimed that the previous hospitalization was unsuccessful and had to be terminated because they discovered that hospital staff were cooperating with her husband’s mistress and released him at night to meet her secretly. She maintained that the woman had even followed them during their holiday trip to Greece. The patient could not remember this but he agreed that it must have had certainly happened. He was completely uncritical in his narration. They both tried to intervene by contacting “the mistress,” who finally wrote to the chancellor of the university and stated that she did not know and had no relationship with Mr. D and complained that she was intruded on by the couple. Shortly afterwards, the patient retired. Since then, Mr. D and Mrs. E were spending almost all their time together and both perceived their relationship very well apart from the presented problem. Mrs. E was interviewed but she refused answering any questions about herself, pretending they were “strictly confidential.” She was not at all motivated to discuss her feelings and expected only an intervention that would prevent her husband from seeing “the mistress” and making financial decisions in her favor (they were not able to present any document as evidence of it).

Mr. D was routinely diagnosed during his stay in the ward. No previous psychiatric morbidity could be found; both their family history was also negative (as stated). He had comorbid arterial hypertension, coronary artery disease, and diabetes, all properly controlled pharmacologically. Due to an untreated erectile dysfunction (ED), he had not had sexual intercourse with his wife or other women for a few years. He was upset about this and did not know that effective treatments of ED were available. He consented to eventual medical treatment that could improve his sexual function. Psychological evaluation made during previous hospitalization (Minnesota Multiphasic Personality Inventory, Benton Test, Wisconsin Card Sorting Test) showed no relevant disorder apart from a decrease in abstract reasoning. A recent brain Computed Tomography scan showed only slight ventricle extension and sclerotic plaques in internal carotid arteries. Other standard examinations were not significant.

In the course of hospitalization, the patient was calm, slightly depressed, once becoming ambivalent about his delusional thoughts but after each of his wife’s daily visits reassured about their correctness. The dose of risperidone was lowered. After only a few days of being in the ward, Mrs. E claimed that “the mistress” was seen in the hospital by the people she hired to observe her husband and she took him out of the ward. Mr. D initially denied the insinuation but later admitted it must have happened if his wife said so. Mrs. E refused to bring the recordings documenting her husband escaping from home with “the mistress” as “the tapes were left in another city and hard to obtain.” During every conversation, she was extremely watchful and suspicious. After initial objections, the couple finally permitted me to contact their son who lived in another country. In a telephone conversation, he stated that for many years he had had very poor contact with his mother and recently the relations with his father had also become much worse. He perceived his mother as definitely ill and remembered that in the past she used to take some kind of psychotropic medication. He was also not motivated for any involvement in his parents’ therapy and did not want to maintain any closer relations with them. Mr. D and Mrs. E lived a relatively solitary life and had no close relations with other people.

After several examinations, the nature of delusional beliefs was discussed with the patient, and he did not resist the suggestion that his wife should be simultaneously treated. However, the next day, Mr. D and Mrs. E declared that Mr. D had to leave the hospital immediately due to his wife’s “unexpected business trip” that was “strictly confidential” and “would last several months.” Mrs. E refused any offer of treatment and convinced her husband to sign himself out of the psychiatric ward. No premises for involuntary hospitalization could be found, so their request had to be respected.

## Discussion

Why is there so limited research on SPD and the prevalence of this phenomenon is so hard to obtain? One possible explanation is that SPD is an extremely rare condition. The other, more convincing reason is that people sharing psychotic disorder are not likely to seek psychiatric treatment due to lack of insight and defending their intimate thoughts unless they break the law or their behavior is exposed to the public. However, couples affected by SPD with sexual delusions may seek for sexual therapy of an alleged sexual abnormality.

This is the first report in the literature on patients with SPD attending a sexologist’s office sharing a delusional belief of one’s infidelity or deviant behavior. The prognosis for treating people with SPD is usually poor because of lack of motivation, poor insight, and defending against separation of affected individuals. If not treated involuntarily, patients often abandon therapy, as in this case.

Presenting negative outcomes allows us to consider therapeutic failures that could be avoided, possible negative consequences as well as legal issues that should be improved in the future. I would like to concentrate on several questions: Was the diagnosis of SPD appropriate and what would be the advice for sexologists seeing such patients? What could underlie the development of such a unique type of delusional system? What limited my approach and did I exhaust therapeutic possibilities in this case? Were there premises for involuntary treatment?

As noted above, most individuals who share psychotic symptoms (folie à deux), both inducers and recipients, do not seek professional care or treatment due to lack of insight. This may be different for individuals with SPD with sexual delusions. In my case, the patient persistently looked for treatment on the inducer’s initiative, related to the delusional belief of sexual pathology of an induced partner. In other words, the belief of the induced partner’s exploitation and unaccepted sexual behavior was one of the core symptoms of delusional disorder. This must be distinguished from Munchausen-by-proxy Syndrome (MbPS), where patients simulate physical or psychological symptoms in a related person. The important difference between MbPS and SPD is the intentional production of symptoms (factitious disorder) in another person and motivation to assume the sick role by proxy in the former, whereas in the latter the symptoms are of clearly psychotic origin.

In the presented case, the psychotic background of reported partners’ behavior seems to be evident and the couple met the criteria of SPD or IDD according to DSM-IV-TR or ICD-10, respectively. No history of psychiatric illness could be found in the recipient, but some evidence on previous psychiatric treatment of Mrs. E was given by her son. Additionally, the origins of psychotic illness in the inducer were hard to obtain due to lack of objective data. Unlike other case reports on SPD, this one contains many more details about the recipient while the information on the inducer was very limited. The explanation for that is that other case reports rely on involuntary treatment conditions or court investigations. Mrs. E was strongly concentrated on sharing her delusional beliefs and allowed to perform all the examinations of her husband, while she was getting very suspicious and resistant to answering questions about herself. She also did not allow Mr. D to disclose any information concerning her. Finally, Mrs. E stated that she worked for the secret service and that her private data were strictly confidential. This was likely to be a part of her delusional system but, in fact, it limited the possibilities of obtaining more data on the inducer’s case. I also did not succeed in establishing therapeutic relation with Mrs. E as she refused to discuss her emotions (apart from expressing anger against “the mistress” and worries about her husband’s mental state) and jumped quickly to talk about possible interventions focused on Mr. D.

In the presented case, the diagnosis of SPD was supported by an unusually close relationship between these individuals, one being dominant (Mrs. E) and other submissive (Mr. D), and their relative isolation from others, including family and non-relatives. Mr. D’s symptoms started to remit when separated from Mrs. E (even when the risperidone dose was lowered), which further confirms the existence of induced psychosis. I therefore predict that a longer separation would benefit the induced individual in the presented case–this could liberate him from induced delusional thoughts and improve his cognitive function. It is consistent with a common observation that induced partner’s delusional symptoms may diminish or disappear when the relationship with the inducer is interrupted. However, according to Shiwach and Sobin (1998), in most cases of affected monozygotic twins, there was only transient or no improvement after the separation. They concluded that blood-related family members who improve on separation are worth following up over time. McNiel, Verwoerdt, and Peak (1972) found that positive outcome may be also less likely in elderly pairs with shared psychosis. This may be attributed also to Gralnick’s subtype C of SPD—folie communiqué (Table 2), where the induced partner develops psychosis after a long period of resistance and symptoms maintain even after separation from the inducer. The presented case was rather closer to Gralnick’s subtype A, apart from the induced partner being here not the younger but the older person (from Gralnick’s classification, only subtypes A and C meet recent diagnostic criteria for SPD as subtypes B and D attribute endogenous psychosis to both partners). Mr. D also revealed other common risk factors of being the recipient of his partner’s delusional beliefs, like suggestibility and passiveness. This might be compared to mild cognitive impairment that was found in his case, quite common for older people with cardiovascular risk factors.

**Table 2:** Classification of Shared Psychotic Disorder by Gralnick (1942)

Subtype of SPD	Description
A (folie imposée)	The dominant person with delusions imposes his or her delusions on a younger and more submissive person. Both persons are intimately associated, and the delusions of the recipient disappear after separation
B (folie simultanée)	The simultaneous appearance of an identical psychosis occurs in two intimately associated and morbidly predisposed individuals
C (folie communiguée)	The recipient develops psychosis after a long period of resistance and maintains the symptoms even after separation
D (folie induite)	New delusions are adopted by an individual with psychosis who is under the influence of another individual with psychosis

It can be supposed that some delusional statements of patients seeing a sexologist could initially seem believable. Until more information comes to light or delusions become more evident or irrational, these cases can be treated as disturbed sexual behavior. It indicates a critical need for establishing the probability of patients’ complaints in similar cases. It is always important to rule out the possibility that events thought to be delusional indeed happened. In the presented case, clearly there was no evidence for any of the sexual practices suspected by the inducing partner. Mr. D suffered from ED that prevented sexual activity with his wife. This could be explained most probably by his comorbidities—cardiovascular disease and diabetes that are common risk factors for male sexual dysfunction (Apostolo et al., 2009; El-Sakka, 2007).

It is interesting whether sexual dysfunction could act here as a “trigger point” for inducing the partners’ delusional ideation about patients’ infidelity, exploitation or deviant behavior. This would correspond with psychodynamic and cognitive reasoning of delusional creation in predisposed individuals. So, a hypothesis about the origins of Mrs. E’s specific psychotic symptoms would be as follows: She had probably been suffering from delusional disorder for many years. Mr. D’s sexual dysfunction combined with Mrs. E’s state of “psychotic readiness” could be a trigger to develop a new delusional system. It is very common that a woman perceives her partner’s ED as a sign that she is not attractive to him anymore or even that he is having an affair with someone else. In the case of a woman suffering from psychotic disorder, this could be easily interpreted and incorporated into a delusional system. These women will be concentrated on stopping an alleged affair rather than treating their partner’s ED. In the case of delusional jealousy, Mrs. E’s belief that her husband was exploited by the mistress against his will might have protected their loving relationship—“he is not unfaithful, but mentally ill, insane.” It is only an assumption, of course. But, in fact, Mrs. E was convinced that they could have a successful marriage and sexual life if only her husband would not meet the mistress (even if confronted with his medical situation as a background of sexual dysfunction). She opposed the idea of treating Mr. D’s ED as it was perceived only as the consequence of sexual exploitation by the mistress. Mrs. E did not blame her husband for the situation, but simultaneously she accurately controlled his stay in the hospital and later even accused the staff of admitting the mistress to meet the patient during the night, which remains consistent with her delusional jealousy.

Mrs. E resisted any kind of examination or intervention focused on her. If she had consented, her therapy could combine antipsychotics with psychotherapeutic approach focused on emotions of sexual dissatisfaction, jealousy, lack of trust and relationship with her husband, who could be simultaneously treated for sexual dysfunction. In the case of Mrs. E’s recovery, separation from Mr. D and his antipsychotic treatment would probably not be necessary. However, if Mrs. E refused therapy, separation would be justified and could benefit her husband’s therapy. It is recommended that separation should last for at least 6 months. It is usually very hard to obtain as both partners are tightly linked to each other and tend to defend the isolation that makes them relatively happy. This was also found in the presented case. Separation would also demand applying the procedure of involuntary hospitalization of one or both partners. Then, only the inducer (Mrs. E) would need antipsychotic therapy. For the recipient (Mr. D), separation and psychoeducation would probably be sufficient for recovery. Similar to many other countries, in Poland involuntary hospitalization can be applied only when a person threatens directly their own or another person’s life or health or is incapable of meeting basic vital needs. In the presented case, there was no evident life nor health threat. Legal regulation does not determine whether psychotic induction can be seen as harming someone’s health when the victim’s insight is lacking. Involuntary hospitalization could be applied when at least one of the patients (inducer or induced partner) would break the law.

Joshi et al. (2006) presented a case of folie à trois where three affected sisters were involuntarily hospitalized and discharged to outpatient care under the provision (ordered by the court) of living in separate counties and prohibited from visiting each other without supervision. However, in that case, the court order was supported by the fact that the affected individuals had committed a penalized crime–burglary, assault and battery with intent to kill, and they were adjudicated not guilty by reason of insanity. Another case of involuntary treatment of a couple sharing psychotic disorder that was charged with the abduction of a woman was described by Newman and Harbit (2010). In other, less threatening cases (like the presented one), such a solution may be impossible because of legal regulations. Moreover, even if separation was applied in the presented case, the prognosis would still be uncertain. If Mrs. E would prove to be resistant to therapy (which often happens in patients with delusional disorder), prolonging the separation could also be harmful, as both partners were living in a relatively happy relationship, even if overwhelmed by a shared delusional system. Further investigation is needed to establish a long-term efficacy of different therapeutic settings in patients sharing delusional beliefs.

### Conclusion

SPD is one of the most confusing and poorly validated psychiatric disorders. Couples sharing delusions that concern sexual behavior are referred to sexual health professionals. The literature on such cases is lacking. This might be due to weak understanding of this relatively rare phenomenon, diagnostic difficulties, and challenging therapy of SPD with frequently negative outcome. SPD in non-consanguineous partners sharing sexual delusions referred to a sexual medicine professional was presented in the literature for the first time. Therapeutic reversal in the presented case was strongly connected with lack of insight, inducer’s resistance, and legal limitations preventing involuntary treatment and separation of the affected individuals. Further research is needed to evaluate the epidemiology and nosological status of SPD. The possibility of implementing more efficient therapy in the future would rely on changes in legal regulations and validation of therapeutic settings.
